# Covalent organic-inorganic polyoxometalate hybrids in catalysis

**DOI:** 10.3389/fchem.2024.1447623

**Published:** 2024-08-29

**Authors:** Tuba Iftikhar, Mali H. Rosnes

**Affiliations:** Department of Chemistry, University of Bergen, Bergen, Norway

**Keywords:** polyoxometalates, hybrid POMs, catalysis, organofunctionalization, class II poms

## Abstract

Polyoxometalates (POMs) are a class of compounds known for the vast range of tunable structures and properties available, leading to applications in areas such as catalysis, energy, and advanced medicine. The ability to covalently functionalize POMs with organic components has been investigated extensively to tune the physical and chemical properties of the resulting hybrid materials. These hybrids, where the organic entity is covalently attached to the POM-core (***Class II*** hybrid POMs) result in a vast library of promising customizable catalytic systems, displaying tunable properties with a high level of synergy between the polyanion and the organic component. A number of ***Class II*** hybrids have been investigated for a wide range of catalytic applications, and here, we give a brief overview of ***Class II*** hybrids of the p-block elements and their applications in catalysis.

## 1 Introduction

Polyoxometalates (POMs) represent an exceptional group of metal-oxo nanoclusters, comprising metal-oxygen-metal linkages, typically constituted of early transition metals (group V or VI) in their highest oxidation states, with a large degree of structural and compositional versatility (Pope and Müller, 1991). POMs are obtained by condensation processes of simple anions in solution *via* careful tuning of the synthetic parameters *i.e.*, concentration, pH, counter-cations, and temperature conditions (Dolbecq et al., 2010; Hutin et al., 2013). Due to the vast range of possible structures available and the possibility to control and fine-tune their properties, POMs are potential candidates for applications in life science (Bijelic et al., 2019), molecular magnetism (Clemente-Juan et al., 2012), and catalysis (Weinstock et al., 2018).

The presence of terminal and bridged oxygen atoms in POMs highlights their intrinsic potential to be applied as inorganic multidentate ligands with versatile topologies. This is particularly evident in the lacunary species, where one or more metal addenda are missing compared to the “plenary” or complete parent polyanion (Long et al., 2006). A variety of organic entities have been covalently grafted to POMs via these lacunary sites, by employing for example, organosilicon functionalization. Another approach is the use of organic alkoxy groups which bond to plenary polyanions like on the Anderson-POM *via* esterification or condensation reactions with protonated bridging oxo groups on the POMs (Chen and Zubieta, 1992). Both these examples are categorized as ***Class II*** hybrids, where the organic and inorganic moieties are linked via covalent or iono-covalent bonds (Dolbecq et al., 2010; Proust et al., 2012). ***Class II*** hybrids provide good chemical control of the bridging unit and hence the interaction between the units, as well as spatial control of the resulting compounds (Kibler and Newton, 2018). Although Class-II hybrids provide enhanced versatility of POM archetypes for tailoring the properties of resulting compounds for specific applications, the covalent or iono-covalent bonds between the POM core and organic ligands are more vulnerable to hydrolysis. Their hydrolytic stability is largely affected by temperature, nature of polyanion, type of organic ligand, and especially extreme pH conditions (Dolbecq et al., 2010). However, studies showed improved hydrolytic stability under certain physicochemical conditions (Hou and Hill, 1993; Al-Sayed et al., 2015; Blazevic et al., 2015; Salazar Marcano et al., 2021; Nowicka et al., 2024).

**FIGURE 1:**
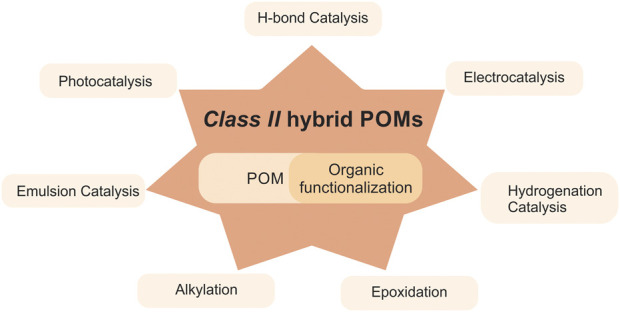
An overview of POMs in catalysis.

POMs have been intensely studied due to their versatile structures and tunability of respective properties, and have been investigated for a large number of catalytic applications, see for example, review by Yang (Wang and Yang, 2015). ***Class II*** hybrid POMs are promising firstly due to the excellent control and rational design of molecular assemblies, secondly, due to their versatility in liquid organic reactions arising from the wide variety of organic groups and the possibility to fine-adjustment the electronic surface states, and finally, for heterogenization purposes where a known catalyst is tethered to a POM-unit simply for heterogenization of the otherwise homogeneous catalyst. Interestingly, such tethering to POMs has also been reported to increase the catalytic activity, highlighting how the nature of the bridge between the two parts is essential for tuning the catalytic activity (Bar-Nahum et al., 2003; Mirkhani et al., 2008a). An overview of ***Class II*** hybrid POMs in catalysis is shown in Figure 1.

One of the key aspects for POMs in catalysis is the ability of polyoxoanions to be reversibly reduced without significant structural deformation, and due to the high number of metal centers present, POMs can undergo multi-electron redox processes. This has led to a large activity investigating POMs as photocatalysts. The visible light absorption of POMs is typically low (it is predominantly in the UV-region), but the visible light absorption can be increased by coupling POMs to suitable photosensitizers (PS), where continuous photo-excited electrons from a PS can be transported rapidly to the POM-core for e.g., photocatalytic hydrogen evolution reactions (HER). It is also reported that this can increase the lifetime of photo-generated carriers in the PS and that the electro-hole pair recombination will be suppressed (Li et al., 2020).

The coupling with photosensitizers in both ***Class I*** and ***Class II*** hybrids can allow for light-driven HER, where reports suggest that the effect is largest when utilizing ***Class II*** hybrids (Luo et al., 2020a). A much studied ***Class I*** hybrid is the tetrabutyl ammonium salt of decatungstate (N(C_4_H_9_)_4_[W_10_O_32_]), with its still-unparalleled reactivity, which is found to be active in light-driven direct hydrogen atom transfer reactions (Waele et al., 2016). ***Class I*** hybrid POMs can affect the catalytic properties of the system by steering for example, the catalyst solubility and even affect the catalysts formal reversible redox potentials by utilizing ionic liquids (Zhao and Bond, 2009). Despite the fact that ***Class II*** hybrid POMs often are more challenging to synthesize, ***Class II*** hybrids have the benefits of being able to tune (I) the electronic structure of the resulting catalyst system, with a reported stronger electronic coupling between the photosensitizer and the POM compared with ***Class I*** (Amthor et al., 2022), (II) the solubility, (III) the number of active sites, and (IV) the Brønsted acid activity (Lachkar et al., 2016). Several review papers have recently been published on hybrid POMs (Dolbecq et al., 2010; Proust et al., 2012; Kibler and Newton, 2018; Gouzerh and Proust, 1998; Proust et al., 2008; Long et al., 2010; Zhang et al., 2012; Ivanova, 2014; Liu et al., 2012; Mirzaei et al., 2014; Nisar and Wang, 2012; Nlate and Jahier, 2013; Ren et al., 2015; Santoni et al., 2014; Song and Tsunashima, 2012; Anyushin et al., 2020; Najafi, 2023). For details on the synthetic approaches and challenges, see for example, reviews by Proust (Proust et al., 2008), Dolbecq (Dolbecq et al., 2010), and Parac-Vogt (Anyushin et al., 2020). This review is aimed at giving a short overview of the ***Class II*** hybrids where the bridge between the polyanion and the organic unit is via p-group elements, and their applications in catalysis.

## 2 Class II hybrid POMs and their application in catalysis

**FIGURE 2:**
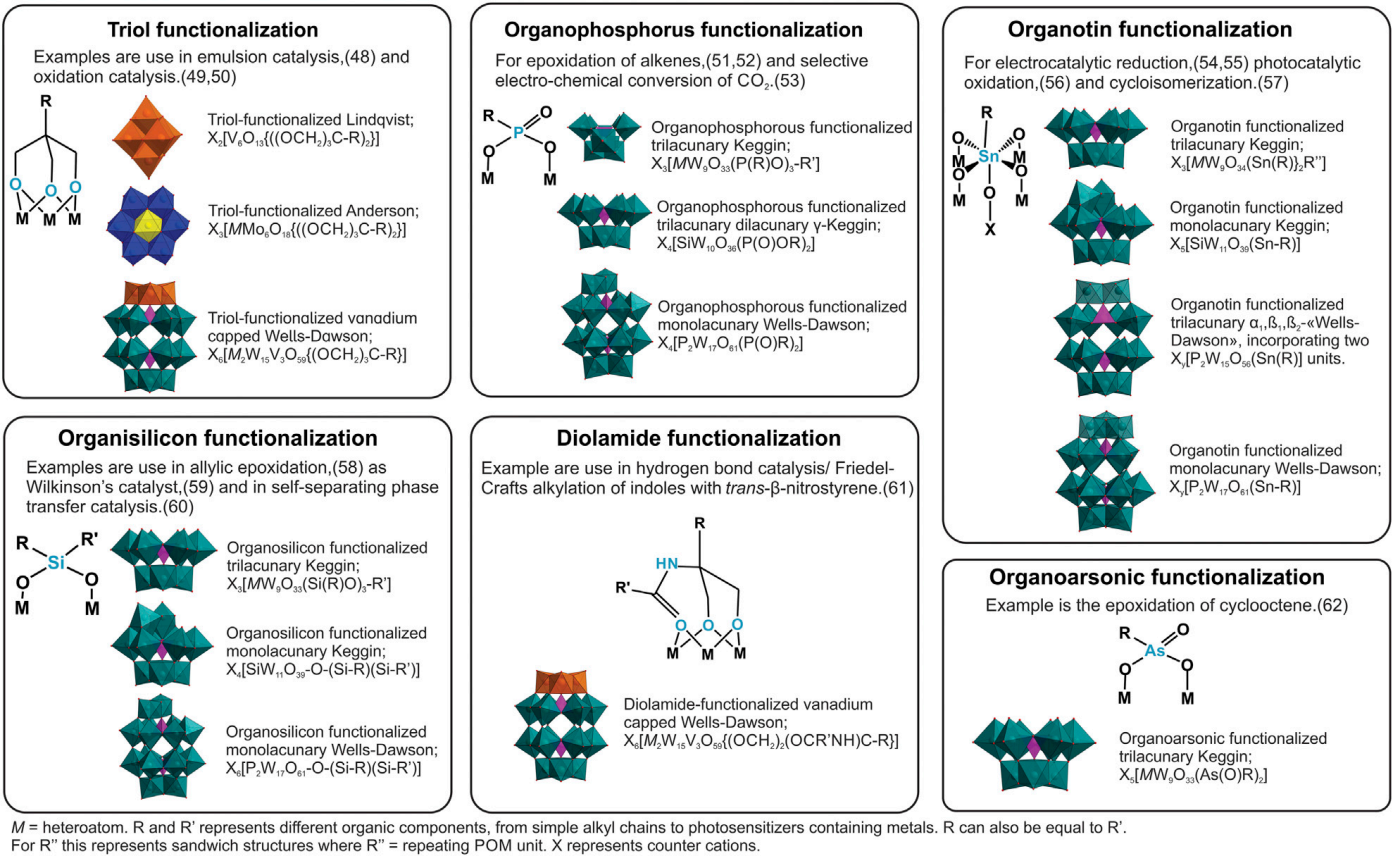
The different types of organo-functionalization discussed here, and relevant POM-archetypes with catalysis examples.

To give a comprehensive overview of ***Class II*** hybrids in catalysis we focus on ***Class II*** hybrids of triol (Liu et al., 1987), imido (Du et al., 1992), organophosphorus (Kwak et al., 1975), organosilicon (Knoth, 1979), organotin (Sazani et al., 2000), and organoarsonic (Barkigia et al., 1981) functionalization. An overview is presented in Figure 2. There are several other examples of ***Class II*** hybrids employing other p-group elements such as organodiazenido (Hsieh and Zubieta, 1986), organogermanium (Sazani and Pope, 2004), and organoantimony (Piedra-Garza et al., 2009) functionalization, but to the best of our knowledge there are no reports on their catalytic activity.

### 2.1 Triol functionalization

The first report on the synthesis of organic-alkoxy derivatives is credited to Knoth in 1981, where both the molybdenum and tungsten versions of the Keggin polyanion were successfully functionalized [PMo_12_O_39_(OMe)]^2−^ and [PW_12_O_39_(OMe)]^2−^ (Knoth and Harlow, 1981). The first report of tripodal-functionalized (triol-functionalized) Anderson was reported in 2002 (Hasenknopf et al., 2002), in 1990 for hexavanadate (Chen and Zubieta, 1990), and in 1993 for the Wells-Dawson (Hou and Hill, 1993). The major exploration of catalytic applications of alkoxy-functionalized covalent hybrids is focused on the Anderson POM, {*M*Mo_6_O_18_((OCH_2_)_3_C-R)_2_}, in addition to some hexavanadate {V_6_O_13_((OCH_2_)_3_C-R)_2_}, and some Wells-Dawson {P_2_V_3_W_15_O_59_((OCH_2_)_3_CR)} examples.

#### 2.1.1 Hybrid Anderson POMs

The electropolymerization of a Mn-Anderson POM bearing two pyridylgroups [MnMo_6_O_18_{(OCH_2_)_3_CNHCO(4-C_5_H_4_N)}_2_]^3−^, with zinc octaethylporphyrins, results in highly ordered hybrid copolymers. The hybrid polymers act as catalysts for the photocatalytic reduction of AgI_2_SO_4_ under visible light and aerobic conditions in the presence of propan-2-ol (Schaming et al., 2010). A tetrabromo-bifunctionalized Mn-Anderson POM has been successfully included in conjugated microporous polymers via Sonogashira–Hagihara cross-coupling with 1,3,5-triethynylbenzene. The polymers exhibited outstanding heterogeneous photocatalytic activities for degrading organic dyes in water, where hydrogen peroxide and singlet oxygen are the primary active catalytic species (Li et al., 2017).

A series of Anderson POM dyads [*M*Mo_6_O_18_{(OCH_2_)_3_CNH_2_}_2_]^3−^, with *M* = Mn^3+^, Fe^3+^, Co^3+^, coupled to an established iridium photosensitizer have been investigated for light-driven hydrogen evolution reaction (HER), where the highest intermolecular excited states were observed for Mn (Schönweiz et al., 2017). The investigation of the photoinduced electron-transfer processes in the dyads found that it was the yield rather than the lifetime of the charge-separated states which determine the catalytic capacity (Luo et al., 2020a). Comparing the structural variation of linkages using an imine (Schiff-base) bond *vs.* a direct C-C bond it was found that the complex with the imine bond increases the HER activity, which might be (partially) attributed to the slightly higher charge-separation yield for the imine-complex (Luo et al., 2020b). The HER activity of a covalently linked Anderson/Pt-complex has also been reported, where the POM acts as a photosensitizer/catalyst-binding site and facilitates light-induced charge-transfer and catalytic turnover (Maloul et al., 2021).

The one-sided tris-functionalized Fe-Anderson POM [[NH_2_C(CH_2_O)_3_]Fe(OH)_3_Mo_6_O_18_]^3-^, has been employed as a molecular heterogenous catalyst for the selective and effective aerobic oxidation of a diverse range of aldehydes to the corresponding carboxylic acids in aqueous media (Yu et al., 2017). A Cr-Anderson POM {CrMo_6_O_18_(OH)_3_[(OCH_2_)_3_CCH_2_OH]}, has been reported as a catalyst for oxidative esterification of alcohols, using low catalyst loading and avoiding expensive ligands in the catalyst system (Wang et al., 2021). The Al-Anderson POM, {AlMo_6_O_18_(OH)_3_[(OCH_2_)_3_CCH_3_]}, has been reported as a catalyst for the oxidation of alcohols to aldehyde, where it was found that added halide ions blocked the catalytic activity by forming hydrogen bonding to the “non-functionalized” side of the POM, which can be reversed by the addition of water (She et al., 2019).

A bi-functionalization on one side of the *β*-Mn-Anderson POM, *β*-{[H_3_NC(CH_2_O)_3_]_2_MnMo_6_O_18_} is reported to behave as a highly active catalyst for selective oxidation of cyclohexanone and cyclohexanol, as well as their mixture, due to the presence of the Mn^3+^ atom site and the topology (Luo et al., 2018).

#### 2.1.2 Hybrid hexavanadate POMs

Both one and two-sided functionalization of the hexavanadate, {V_6_O_13_((OCH_2_)_3_CCH_2_OH)((OCH_2_)_3_CCH_2_OOC(CH_2_)_16_CH_3_)} and {V_6_O_13_((OCH_2_)_3_CCH_2_OOC(CH_2_)_16_CH_3_)_2_}, have been found to be highly efficient emulsion catalysts in oxidation desulfurization processes (Yin et al., 2012). A derivative containing free carboxyl groups, {V_6_O_13_((OCH_2_)_3_CCOOH)_2_}, formed POM-based coordination polymers upon reaction with zinc ions (Bayaguud et al., 2016). The resulting 2D nanosheets demonstrated high catalytic activity for the aerobic oxidation of propanethiol to its corresponding disulfide under mild conditions. A Lindqvist POM attached to porphyrin units coordinated to Zn; {V_6_O_13_{C_61_H_58_N_5_O_4_Zn}_2_}, is reported to have an effective photocatalytic activity for degrading the water-soluble pollutant, Rhodamine B (RhB), under visible-light illumination. The activity is ascribed to the possible energy and electron transfer between the POM cluster and the porphyrin (Zhu et al., 2020).

#### 2.1.3 Hybrid Wells-Dawson POMs

A norbornene monomer containing organofunctionalized {P_2_V_3_W_15_O_62_} POM shows that the catalytic activity of the hybrid for the oxidation of a sulfide was retained when the same hybrid-containing monomer was incorporated in a norbornene-type polymer (Miao et al., 2014). A Cu–dipyridylamine tethered to a Dawson-type {P_2_V_3_W_15_O_62_} POM shows photo-redox properties, where the complex exhibit the ability to store up to three reducing equivalents upon irradiation with visible light. Moreover, its ability to catalytically photogenerate CF_3_ radicals, in the presence of Togni’s reagent is demonstrated (Wang et al., 2023).

### 2.2 Organotin functionalization

Organotin functionalized derivatives of lacunary POMs were first reported by Knoth, with several tungsten-based Keggin-type POMs, such as {SiW_11_O_39_} functionalized with R_2_SnCl_3_ to give {SiW_11_O_39_(SnR)} (Knoth, 1979).

#### 2.2.1 Hybrid Wells-Dawson POMs

A tetra (carboxyethyltin)-decorated POM, {K_2_Na_2_{Sn(CH_2_)_2_COO}_4_(H_2_O)_2_{WO_5_(H_2_O)}(P_2_W_15_O_56_)_2_}, was reported as an electrocatalyst for the reduction of H_2_O_2_. Electrochemical studies demonstrated that it is the reduced hybrid POM species that are electrocatalytically active in the reduction (Zhang et al., 2010). A sandwich-type carboxyethyltin functionalized Keggin POM [K_3_{Sn(CH_2_)_2_COO}_2_(A-*α*-PW_9_O_34_)_2_]^11−^, can function as an electrocatalyst for the reduction of H_2_O_2_, showing enhanced catalytic performance for the oxidation of cyclohexanol compared to its parent species, with good recyclability without the loss of catalytic activity (Yang et al., 2013). An estertin derivative of tungstoarsenate Keggin, {(W_4_O_16_){Sn(CH_2_)_2_COO}_4_(B-*α*-AsW_9_O_33_)_2_}, is reported to act as an efficient, eco-friendly, green catalyst for cyclohexanol oxidation and photocatalytic degradation of RhB, and for H_2_O_2_ detection (Ji et al., 2016).

Catalytically active gold complexes such as [AuPPh_3_]^+^ can be tethered to the Wells-Dawson POM, {P_2_W_17_O_61_{Sn(CH_2_)_2_CO}. The resulting catalysts extend the scope of the cycloisomerization of allenols to substrates that are extremely sensitive to dehydration, as well as stabilizing the cationic gold active sites, which lead to the recycling of the hybrid gold-POM catalysts (Dupré et al., 2012). The Keggin POM functionalized with a photosensitive organotin moiety {SiW_11_(Sn(CH_2_)_2_HCNC_16_H_9_)O_39_} has been reported as an eco-friendly, visible light photocatalyst for aerobic oxidation of sulfides to sulfoxides in good yield at low cost. The covalent tethering significantly reduced the band gap of POM, allowing it to absorb visible light and to conduct catalytic reactions under visible light (Karimian and Zangi, 2021).

### 2.3 Organosilicon functionalization

Organosilicon functionalization is reported for only certain polyoxotungstate versions of the Wells-Dawson and Keggin based structures; {XW_9_O_33_}, {XW_10_O_38_}, {XW_11_O_39_} and the {X_2_W_17_O_61_}. Knoth et al. first reported the reactions of RSiCl_3_ (R = C_2_H_5_, C_6_H_5_, NC(CH_2_)_3_, C_3_H_5_) with {SiW_11_O_39_} (Knoth, 1979).

#### 2.3.1 Hybrid Keggin POMs

The first metallosalen-type ligands anchored on the Keggin POM {SiW_11_O_39_} were reported in 2003 (Bar-Nahum et al., 2003). Several {M(salen)-SiW_11_O_39_} hybrids (M = Co, Fe, Cu, Ni, etc.) followed (Mirkhani et al., 2008a; Mirkhani et al., 2007; Mirkhani et al., 2008b; Moghadam et al., 2010a; Moghadam et al., 2010b), which are reported to exhibit strong electronic effects attributed to the attachment of the metallosalen complex to the POM, leading to an intramolecular charge transfer. This effect was successfully employed in homogeneous (Mirkhani et al., 2007; Mirkhani et al., 2008b; Moghadam et al., 2010b; Karimian et al., 2014) and heterogeneous catalysis (Mirkhani et al., 2008a; Moghadam et al., 2010a) of alkenes with H_2_O_2_ where the hybrid POM shows higher catalytic activity than the metallosalen complexes alone. 

A study of alkene epoxidation catalyzed by [*α*-B-SbW_9_O_33_(^*t*^BuSiO)_3_Ti(O^*i*^Pr)]^3–^ highlights how the allylic epoxidation occurred at the molecular level with aqueous solutions of H_2_O_2_. Two modified catalysts, i.e, [*α*-B-SbW_9_O_33_(*R*SiO)_3_Ti-(OiPr)]^3–^ (R = ^*i*^Pr, and ^*n*^Pr), were able to epoxidize both functionalized and nonfunctionalized substrates (Solé-Daura et al., 2020).

The Wilkinson’s catalyst (Rh(I)Cl(PPh_3_)_3_) inspired POM, {SiW_11_O_39_[O(SiCH_2_CH_2_PPh_2_)_2_PPh_3_Rh(I)Cl]}, has been employed as an effective, recyclable catalyst for the hydrogenation of alkenes under monophasic and aqueous biphasic reaction conditions (Bar-Nahum and Neumann, 2003). The {Pd(salen)–SiW_11_O_39_} was investigated for the Suzuki cross-coupling reaction in EtOH/H_2_O under mild reaction conditions, where the hybrid derivative showed greatly improved catalytic activity. For the coupling of aryl bromides and iodides with both deactivated and sterically hindered aryl-boric acids, both high levels of reactivity and turnover numbers were obtained (Tong et al., 2014).

An anthracene functionalized {SiW_11_O_39_} showed good photocatalytic activity for the oxidation of alcohols to carbonyl compounds under aerobic conditions, with good yields (Karimian et al., 2014). Ti^IV^ complexes, i.e, [XW_9_O_34–*x*_ (^*t*^BuSiO)_3_Ti(O^*i*^Pr)]^3−^ (X = P, *x* = 0; X = Sb, *x* = 1,2), are reported as relevant soluble models for heterogeneous titanium silicalite epoxidation catalysts, showing high selectivity in allylic alcohol epoxidation (Zhang et al., 2018a). A silanol-decorated POM, [SbW_9_O_33_(^*t*^BuSiOH)_3_]^3−^, can stabilize a low-valent d^2^-vanadium^III^ ion in a trigonal coordination environment to aid its distribution on silica or anatase surfaces, resulting in the formation of a vanadium^III^ POM i.e, [SbW_9_O_33_(^*t*^BuSiO)_3_V(tetrahydrofuran)]^3-^, where the V^III^ entity behaves as a putative intermediate in a Mars–van Krevelen type mechanism for partial oxidation of light alcohols (Zhang et al., 2018b). Amphiphilic {PW_11_O_39_-O-(Si(CH_2_)_n_CH_3_)_2_} (n = 2, 7, 11), has been reported, arguing that it is a suitable catalyst for the oxidation of benzyl alcohol at room temperature (Bai et al., 2020).

Sulfonic-acid functionalized POMs [SiW_11_O_39_–O(SiC_3_H_6_SO_3_H)_2_)]^4–^ and [SiW_11_O_39_–(O(SiC_8_H_8_SO_3_H)_2_)]^4–^, alter the behavior of [SiW_11_O_39_]^8–^ as an electron-absorbing group to promote H^+^ release and enhance acidity by decreasing the electron density of O–H on the surface of sulfonic acid groups. The π–π interactions of the benzene ring enervated the dissociation of H^+^ and displayed weak acid-catalyzed hydroxyalkylation and alkylation characteristics (Lian et al., 2022).

Nanoparticles based on alkyl-thiol functionalized Keggin POM [SiW_11_O_40_(SiCH_2_CH_2_CH_2_SH)_2_]^4–^, were employed as binary catalysts for the aerobic oxydehydrogenation of reactive allylic tertiary carbon-hydrogen bonds i.e., vinylcyclohexene and vinylcyclohexane to styrene, where the hybrid POM stabilized the Pd-nanoparticles (De Bruyn and Neumann, 2007). Organosilyl-POMs have also contributed to catalytic photooxygenation in water under heterogeneous conditions (Bonchio et al., 2004), in catalyzing C–C cross-coupling and aromatic dehalogenation (Berardi et al., 2010), in oxidative desulfurization of organic sulfides, (Carraro et al., 2012), and as oxidation catalysts with H_2_O_2_ under microwave irradiation for diverse substrates (Carraro et al., 2006). Finally, by covalent modification with graphitic carbon nitride the O_2_ adsorption of [SiW_11_O_39_]^8−^ is enhanced, and exhibits excellent activity in photocatalytic H_2_O_2_ production, stabilizing the formed H_2_O_2_ under sunlight irradiation (Zhao et al., 2018).

#### 2.3.2 Hybrid Wells-Dawson POMs

A catalytically active Wells-Dawson POM, the {*α*_2_-P_2_W_17_O_61_(SiC_6_H_4_CH_2_N_3_)_2_O}, has been covalently attached to a microporous resin via click chemistry, and the resulting resin was evaluated for the oxidation of tetrahydrothiophene, with good recyclability and reusability without any detectable loss of catalytic activity nor leakage of inorganic components (Xiao et al., 2013).

The covalent tethering of [P_2_W_17_O_61_]^8−^ with sulfonic acid-based organosilane entities resulted in a catalyst with improved esterification efficacy, owing to effective interactions between substrates and the catalyst. The ingrained emulsification–precipitation cycle induces excellent self-recycling properties in the catalytic system, leading to facile and low-cost catalyst recovery at high yields (Lian et al., 2020).

### 2.4 Diolamide functionalization

The first report of a diolamide functionalized POM was on the Dawson type with 2-amino-2-ethyl-1,3-propanediol [P_2_V_3_W_15_O_59_{(OCH_2_)_2_C(Et)NHCOCH_3_}]^5-^, introducing a new family of acidic POMs, in which the acidity of an amide proton is exalted upon grafting to the vanadium-oxide crown (Li et al., 2009).

By using the diolamide functionalization approach to insert tertiary amides and carbamates it was found that these Dawson-based hybrids showed catalytic activity for the oxidation of sulfides with high selectivity (Oble et al., 2011), and because the electron-withdrawing character of the polyanion increases the acidity of the amide proton, the hybrid can be used as a Brønsted organo-catalyst and for the hydrogenation of quinolines (Lachkar et al., 2016). Picolinic amide derivative-functionalized Dawson POM, generating stable palladacycles, is reported as a catalyst for Heck coupling (Riflade et al., 2013). and as an electrophilic catalyst in the allylation of aldehydes and imines (Riflade et al., 2014). Later, it was found that the insertion of a urea-carbonyl into the POM activates the urea toward hydrogen-bond catalysis. The H-bond catalysis of the hybrid POM was tested for Friedel-Crafts arylation of trans-β-nitrostyrene, where it showed improved overall efficiency due to the electron-withdrawing nature of POM scaffold and the catalyst could be recycled several times (Vilona et al., 2021).

### 2.5 Organophosphorus functionalization

Organophosphorus derivatives were first synthesized by Hill et al. by reaction between the monovacant species *α*-[SiW_11_O_39_]^8-^ or *α*-[PW_11_O_39_]^7-^ with PhPOCl_2_, resulting in the formation of the hybrids *α*-[(PhPO)_2_SiW_11_O_39_]^4-^ and *α*-[(PhPO)_2_PW_11_O_39_]^3-^, respectively (Kim et al., 1992).

#### 2.5.1 Hybrid Keggin POMs

Several organophosphrous (and organosilicon) hybrids have been screened as oxidative catalysts with H_2_O_2_ under microwave (MW) irradiation, where it was found that [*γ*-SiW_10_O_36_(PhPO)_2_]^4-^ was the best-performing catalyst for a broad scope of reactions; from the epoxidation of terminal and internal double bonds and sulfoxidation to oxygen transfer to electron-deficient substrates such as sulfoxides (Carraro et al., 2006). H_2_O_2_-based epoxidation of internal and terminal double bonds in hydrophobic ionic liquids was also reported for [*γ*-SiW_10_O_36_(PhPO)_2_]^4-^, with selectivity >99% and up to 200 turnovers per minute under MW irradiation (Berardi et al., 2007). The first example of a tri-phosphopohrous functionalized polyoxotungstate, K_4_H_6_[H_4_{(AsW_9_O_33_)Zn(H_2_O)W_5_O_11_(N(CH_2_PO_3_)_3_)}_2_(μ_2_-O)_2_], was evaluated as a catalyst for alkene epoxidation with H_2_O_2_ in acetonitrile (Huo et al., 2015). Organophosphorus derivatives of the trivacant {AsW_9_O_33_} POM have also been reported as an efficient catalyst for the epoxidation of cyclooctene and cyclohexene with H_2_O_2_ (see section 2.6) (Bentaleb et al., 2015; Makrygenni et al., 2020). Three sandwich-type organophosphorous-functionalized clusters {[Ni(H_2_O)_5_]_*x*_(AsW_6_O_21_)_2_[Ni(OOCCH_2_NCH_2_PO_3_)_2_]_3_} (*x* = 0, 1, 3), exhibited efficient catalytic performance for the oxidation of allylic alcohols to epoxides in water with H_2_O_2_ (Xu et al., 2018).

#### 2.5.2 Hybrid polyoxomolybdats

A crown-shaped 36-molybdic-18-phosphorous POM, where copper was grafted into the polyanions via the amino trimethylene phosphonic acid-based unit, was tested as a heterogeneous catalyst for selective oxidation of sulfides to sulfoxides with impressive catalytic and selective performances after heat treatment (Xu et al., 2020a). Later, the (NH_4_)_17_Na_7_H_12_ [Co(H_2_O)TeMo_6_O_21_{N(CH_2_PO_3_)_3_}]_6_·42H_2_O, was reported as the first polyoxomolybdate-based heterogeneous catalyst for Knoevenagel condensation with efficient catalysis and good recyclability (Xu et al., 2020b). The hybrid polyoxomolybdate (NH_4_)_18_Na_7_H_11_[Zn(H_2_O)TeMo_6_O_21_{N(CH_2_PO_3_)_3_}]_6_·23H_2_O have been evaluated for the catalytic oxidation of thioanisole to sulfoxides (Kong et al., 2021a), whilst the (NH_4_)_6_Na_3_H_13_[TeMo_10_O_37_{CoMo_2_O_6_((O_3_P)_2_C(O)(CH_2_)_3_NH_2_)}_4_]·11H_2_O have been employed as heterogeneous catalysts for the Knoevenagel condensation reaction (Kong et al., 2021b).

#### 2.5.3 Hybrid Wells-Dawson POMs

K_6_[P_2_W_17_O_57_(PO_5_H_5_C_7_)_2_]**⋅**6C_4_H_9_NO has been reported with enhanced redox behavior and photochemistry compared to its purely inorganic counterpart, where it was found that the hybridization of the POM with electron-withdrawing moieties reduced the HOMO–LUMO gap. This facilitated direct visible-light photoactivation, establishing a simple, cheap, and effective approach for the generation of visible-light-activated hybrid nanomaterials (Cameron et al., 2017). A range of organophosphorous-based assemblies of [P_2_W_17_O_61_]^10−^ were explored for fine-tuning of the electron-withdrawing properties in covalently bonded phosphonate groups, providing an orbital engineering approach for tuning the visible-light photocatalytic activity of the hybrid POM system (Fujimoto et al., 2017).

A photochemical molecular dyad composed of a ruthenium-complex photosensitizer covalently linked to a Dawson POM, α-[P_2_W_17_O_61_]^10−^, has been reported as an electron-storage site and hydrogen-evolving catalyst, with on-demand hydrogen release made possible by the addition of a proton donor to the dyad solution (Amthor et al., 2022). A bis-Ru-polypyridyl substituted hybrid POM, {(Ru(dmbpy)(ppt)Cl)_2_P_2_W_17_O_57_}, where dmbpy = 4,4′-dimethyl-2,2′-dipyridyl, and ppt = 4′-(4-phosphonophenyl)-2,2′:6′,2″-terpyridine), enabled the selective electrochemical conversion of CO_2_ to H_2_/CO or formic acid in the presence of a proton donor in solution state. It was demonstrated that the choice of the obtained C1 feedstocks can be controlled by managing the acidity of reaction media (Kuramochi et al., 2023).

### 2.6 Organoarsonic functionalization

The first report of structurally characterized organoarsonic functionalized POM was disclosed by Pope’s group in 1976 (Kwak et al., 1976).

Organoarsonic functionalization of the tri-lacunary Keggin with free amine groups, {[PW_9_O_34_{As(O)(C_6_H_4_NH_2_)}_2_}, as well as the related organophosphorous functionalized {[AsW_9_O_34_{P(O)(CH_2_CH_2_CO_2_H)}_2_}, were shown to be efficient catalysts for cyclooctene epoxidation with aqueous H_2_O_2_ at room temperature in homogeneous conditions (Bentaleb et al., 2015). The hybrids were anchored to silica supports and again employed for the same catalytic reaction resulting in conversions in the range 19%–97% after 24 h. The difference is ascribed to the POM-organization on the silica-supports. {[PW_9_O_34_{As(O)(C_6_H_4_NH_2_)}_2_} was again reported on mesoporous silica supports but using phenylene diisothiocyanate as a cross-linking agent. The catalytic activity was evaluated in the epoxidation of cyclooctene with aqueous H_2_O_2_ in acetonitrile at 50 °C (cyclooctene conversion of 97% for amine-{PW_9_} and 43% for the diisothiocyanate-{PW_9_}), which was reduced when grafted on to the silica (Makrygenni et al., 2019).

The three POMs B,*α*-{NaHAsW_9_O_33_{P(O)R}_2_} with -tBu and -CH_2_CH_2_CO_2_H, and A,*α*-{NaHPW_9_O_34_{As(O)p-C_6_H_4_NH_2_}_2_} (Makrygenni et al., 2020), has been studied for their catalytic oxidation properties for the homogeneous epoxidation of limonene with H_2_O_2_ at room or low temperature (4 °C), as well as cyclooctene and cyclohexene. The organoarsonic functionalized POM showed a lower efficiency for the conversion of limonene, but it is not apparent if this is due to the swap of heteroatom, or the different functionalization.

## 3 Summary and future perspectives

Herein we have provided an overview of ***Class II*** hybrid POMs in catalysis, highlighting the different types of functionalization and types of POMs that have been investigated. ***Class II*** hybrid POMs show a wide range of catalytic applications, highlighting the feasibility and flexibility of this class of materials. It is apparent that hybrid POMs have a large potential for a wide range of catalytic applications, for example, in photocatalysis. One of the major benefits for using the ***Class II*** hybrids for photocatalysis is the increase in the absorption of visible light, and this outweighs the known challenges for synthesizing more complex ***Class II*** hybrids. There are some highly successful ***Class I*** hybrids used in photocatalysis, and in particular the decatungstate POM, which have the benefits of a simpler synthetic strategy, which show high stability and the ability to undergo reversible multi-electron processes. However, for developing more efficient photocatalysts with tuenability and flexibility under visible-light, developing catalysts based on ***Class II*** hybrid POMs is an exciting and promising field. A more systematic approach comparing types of POMs, the nature of the anchoring, and properties of the attached organic entity would be beneficial for the future development of hybrid POMs in catalysis. This, in parallel with theoretical studies holds promise for future application of hybrid POMs in catalysis.
